# Identification of candidate sex hormone-associated genes and immune infiltration characteristics in osteoarthritis based on bioinformatics analysis and machine learning

**DOI:** 10.1371/journal.pone.0351556

**Published:** 2026-06-12

**Authors:** Yishu Wang, Ling Zhu, Shuna Jin, Yuhan Wang, Zhaoxiang Zeng, Yunzhou Zuo, Xingliang Xiang, Xugui Li, Rongzeng Huang, Chengwu Song

**Affiliations:** 1 School of Pharmacy, Hubei University of Chinese Medicine, Wuhan, China; 2 The Affiliated Hospital of Wuhan Sports University, Wuhan, China; 3 School of Basic Medical Sciences, Hubei University of Chinese Medicine, Wuhan, China; 4 Hubei Shizhen Laboratory, Wuhan, China; 5 School of Acupuncture-Moxibustion and Orthopedics, Hubei University of Chinese Medicine, Wuhan, China; Università degli Studi della Campania, ITALY

## Abstract

**Background:**

Sex hormones play critical roles in the pathogenesis and progression of osteoarthritis (OA), yet the hormone-related molecular networks remain poorly defined. This study aimed to identify candidate sex hormone-associated genes in OA and to explore their potential functional enrichment and immune-related characteristics using bioinformatics analysis.

**Methods:**

OA gene expression data were obtained from the GEO database and integrated with candidate sex hormone-associated genes retrieved from GeneCards. The R package “limma” was then used to identify differentially expressed genes (DEGs) and sex hormone-associated DEGs (SADEGs). OA-associated SADEGs, termed OA-SADEGs, were selected using weighted gene co-expression network analysis (WGCNA), and their potential biological functions and pathways were explored by GO and KEGG enrichment analyses. Hub genes were identified using three machine learning models. xCell analysis was used to estimate immune infiltration and its associations with hub genes, and hub gene expression was further evaluated in external datasets and peripheral blood samples.

**Results:**

We identified 32 sex hormone-associated genes in OA, enriched in extracellular matrix remodeling, receptor signaling, and antigen presentation pathways. Three candidate hub genes (LOXL1, HLA-DRA, and CYBB) were consistently upregulated in OA and showed significant correlations with immune infiltration scores. xCell analysis identified 13 differentially enriched immune cell types, of which three were associated with hub genes. External dataset analysis and peripheral blood qRT-PCR showed upregulation of LOXL1, HLA-DRA, and CYBB in OA samples.

**Conclusion:**

This study integrated bioinformatics and immune analyses to identify candidate sex hormone-associated genes in OA. These findings provide associative bioinformatics evidence for sex hormone-associated molecular features in OA.

## Introduction

OA, the most prevalent form of degenerative joint disease, is widely recognized as a primary source of chronic pain in middle-aged and elderly individuals. It causes joint pain, structural deformities, and impaired function, and is also associated with an increased risk of cardiovascular events and fractures. Globally, OA affects more than 7% of the population, corresponding to approximately 528 million individuals, and ranks as the fifteenth leading cause of years lived with disability (YLDs), accounting for 2.2% of total global YLDs [1]. OA exhibits significant sex differences, with a notably higher prevalence and increased severity of pain among women aged over 50 years, particularly in postmenopausal populations [2]. Female patients generally experience greater pain intensity, faster disease progression, and more severe functional impairment than male patients [3]. These sex-specific disparities in OA are likely attributable to multiple factors, including sex hormone-related changes, particularly age-related declines in estrogen levels, anatomical differences, enhanced synovial inflammatory responses, and differences in central sensitization and biomechanical factors [4].

Sex hormones play a crucial role in the pathogenesis of OA, influencing its development through both direct effects on tissues and indirect effects on inflammatory and immune pathways. These hormones act on joint tissues—including cartilage, subchondral bone, and synovium—via diverse signaling mechanisms, regulating extracellular matrix metabolism, cellular apoptosis and proliferation, as well as inflammatory responses [5]. Major joint cell types, including chondrocytes, osteoblasts, and osteoclasts, express sex hormone receptors and respond to both systemic and local hormonal changes [6]. At the molecular level, sex hormones have been found to regulate key signaling pathways such as Wnt/β-catenin and TGF-β/Smad, thereby contributing to cartilage homeostasis, immune modulation, and bone metabolism [7–9]. Despite increasing interest in the role of sex hormones in OA, current research predominantly focuses on isolated hormonal effects or individual signaling pathways. However, a comprehensive understanding of the hormone-associated molecular networks involved in OA progression remains limited.

Bioinformatics is an interdisciplinary field that integrates biological data with computational methods, enabling efficient analysis, storage, and interpretation of high-throughput biological information [10]. This capability is critical for uncovering novel insights into OA pathogenesis and identifying disease-related biomarkers. Machine learning further strengthens this strategy by enabling pattern recognition and feature prioritization for biomarker discovery and disease classification [11]. The integration of bioinformatics and machine learning provides a useful exploratory framework for identifying candidate sex hormone-associated molecular features in OA. In this study, we applied bioinformatics and immune infiltration profiling to identify differentially expressed candidate sex hormone-associated genes in OA. These candidate genes were then subjected to functional enrichment analysis, hub gene selection, and validation in external datasets. Immune cell correlation analyses were performed to evaluate their immune-related associations, and qRT-PCR further assessed hub gene expression in peripheral blood samples.

## Materials and methods

### Screening and processing of OA-related datasets and collection of sex hormone-associated genes

**Fig 1:**
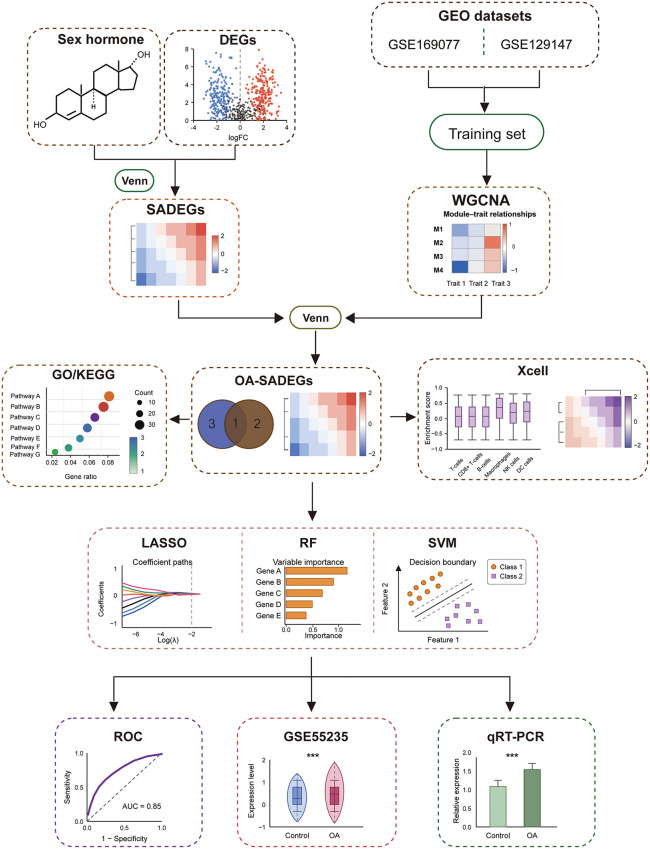
Flowchart showing the comprehensive analysis of sex hormone-associated genes in osteoarthritis.

We downloaded three datasets (GSE169077, GSE129147, GSE55235) from the Gene Expression Omnibus database (GEO) (https://www.ncbi.nlm.nih.gov/geo/) for OA-related gene expression analysis and hub gene identification. GSE169077 was based on the GPL96 [HG-U133A] Affymetrix Human Genome U133A Array and contained 5 normal and 6 OA samples. GSE129147 was based on the GPL15207 [PrimeView] Affymetrix Human Gene Expression Array and contained 9 normal and 10 OA samples. GSE55235 was based on the GPL96 [HG-U133A] Affymetrix Human Genome U133A Array and contained 10 normal and 10 OA samples. GSE169077 and GSE129147 were used as the training set and GSE55235 as the test set. The dataset identifiers were converted using the “org.Hs.e.g.,db” R package. Quantile normalization was performed across the combined GEO datasets to ensure comparable expression distributions. Batch effects between the training datasets were corrected using the “sva” R package, and the effectiveness of batch correction was evaluated by visual inspection of sample distributions before and after correction (S1 Fig). Sex hormone-associated genes were initially collected from the GeneCards database (https://www.genecards.org/) using multiple sex hormone-related keywords, including “sex hormone”, “estradiol”, “estrogen”, “testosterone”, “progesterone”, “follicle-stimulating hormone”, and “luteinizing hormone”, combined using the Boolean operator “OR”. Genes with a relevance score > 1 were retained as preliminary candidate genes. The flowchart depicting the study design is shown in Fig 1.

### Identification of SADEGs

DEGs were identified using the R package “limma”, with |Log2FC| > 2 and *p* < 0.05 as the selection criteria. A heat map and volcano plot were used to visualize the DEGs, and a Venn diagram was created to identify the overlap between DEGs and sex hormone-associated genes, termed SADEGs.

### WGCNA and screening for OA-SADEGs

WGCNA identifies gene modules with coordinated expression by constructing co-expression networks, providing a systems-level view of gene expression data [12]. We used the R package “WGCNA” for WGCNA analysis. Genes ranked in the top 50% by average standard deviation were selected for constructing the gene co-expression network. First, Pearson’s correlation coefficient was used to calculate gene-to-gene correlations within each group. Next, the “pickSoftThreshold” function was used to determine the soft threshold, which converted gene correlations into weighted values to construct a weighted co-expression network. Then, hierarchical clustering was used to group genes with similar expression patterns into modules, each assigned to a unique color, with a minimum module size of 30 and deepSplit set to 3. We used the correlation coefficient and *p* to identify the modules most strongly associated with OA traits. Genes within these modules were filtered using the criteria MM > 0.8 and GS > 0.1 to select strongly correlated genes. The strongly correlated genes selected from the OA-associated modules were intersected with SADEGs to define OA-SADEGs.

### Functional enrichment analysis of OA-SADEGs

GO and KEGG pathway analyses of OA-SADEGs were performed using the R package “clusterProfiler”, with *p* < 0.05 considered statistically significant.

### Machine learning for hub genes

Three machine learning algorithms, namely least absolute shrinkage and selection operator (LASSO) regression, support vector machine-recursive feature elimination (SVM-RFE), and random forest (RF), were used to identify hub genes from OA-SADEGs. For LASSO regression, ten-fold cross-validation was used to determine the optimal λ value with the minimum classification error. LASSO regression was performed using the R package “glmnet” and was primarily used to select feature variables from OA-SADEGs. RF, a machine learning algorithm based on decision trees, was applied to screen genes using “IncNodePurity”, implemented via the R package “randomForest”. SVM-RFE, a machine learning approach based on SVM, was employed to identify optimal variables by iteratively removing feature vectors. Ten-fold cross-validation with five replicates was performed using the R package “caret” to identify the optimal feature subset with the lowest RMSE. The genes selected by the three machine learning algorithms were intersected using a Venn diagram to identify the final hub genes.

### Evaluation of the discriminative ability of hub genes

ROC curves were used to assess the discriminative ability of the hub genes, and their expression levels were compared between OA and control samples in both the training and test sets. An AUC > 0.7 in the ROC analysis was considered to indicate potential discriminative ability, while a *p* < 0.05 in the expression analysis was considered statistically significant.

### xCell immune infiltration analysis

xCell is a gene signature–based method used to compute and visualize relative infiltration scores of 64 immune and stromal cell types from microarray-derived gene expression data. Statistical significance was defined as *p* < 0.05. Spearman correlation analysis was performed, with r > 0.7 considered to indicate a strong correlation (*p < 0.05; **p < 0.01; ***p < 0.001). To explore immune-related associations of hub genes in OA, we analyzed the correlations between hub genes and 13 infiltrating immune cell types, using *p* < 0.05 and r > 0.7 as thresholds. To further examine the consistency of the immune infiltration patterns, an independent external dataset was additionally analyzed using xCell, and the major immune cell signatures identified in the training set were re-evaluated.

### qRT-PCR validation

Peripheral blood samples from 20 patients with osteoarthritis (OA) and 20 individuals without OA were collected at Wuhan Orthopedics Hospital of Integrated Traditional Chinese and Western Medicine (Affiliated Hospital of Wuhan Sports University), Wuhan, China. OA was diagnosed based on clinical and radiographic criteria. Individuals without clinical or radiographic evidence of OA were included as controls. Exclusion criteria included severe systemic diseases, bone metabolic disorders, pregnancy, and other major medical conditions. All participants were adults (≥18 years). Female participants were postmenopausal, and male participants were age- and BMI-matched to the female participants. This study was approved by the Ethics Committee of Wuhan Orthopedics Hospital of Integrated Traditional Chinese and Western Medicine (Affiliated Hospital of Wuhan Sports University) (No. 672HREC20250310-L16). The study was conducted in accordance with the Declaration of Helsinki. All samples were collected within the ethics-approved study period. Written informed consent was obtained from all participants.

Total RNA was extracted from blood samples using the Blood RNA Extraction Kit (G3636-50T). Complementary DNA was synthesized via reverse transcription with the SweScript All-in-One RT SuperMix for qPCR (One-Step gDNA Remover) (G3337-50), and qRT-PCR was subsequently performed using 2 × Universal Blue SYBR Green qPCR Master Mix (G3326-05). Three technical replicates were maintained for each sample. GAPDH was used as the reference gene, and relative gene expression levels were quantified using the 2 − ΔΔCt method. Statistical analysis was performed using a t-test, with *p* < 0.05 considered statistically significant.

## Results

### Identification of DEGs and SADEGs

**Fig 2:**
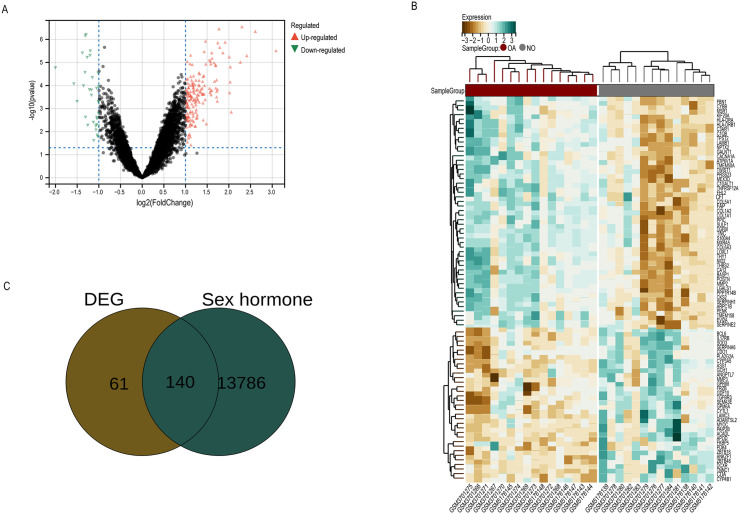
Identification of DEGs and SADEGs. **(A)** Volcano plot of DEGs. Red indicates upregulated DEGs, and green indicates downregulated DEGs. **(B)** Heatmap showing the top 50 DEGs. **(C)** Venn diagram showing the intersection of DEGs and sex hormone-associated genes.

Differential expression analysis identified 201 genes, comprising 168 upregulated and 33 downregulated genes in osteoarthritis. Volcano plots were generated to visualize these genes, with upregulated genes shown in red and downregulated genes shown in green (Fig 2A). A heatmap highlighted the top 50 most differentially expressed genes (Fig 2B), and a total of 140 SADEGs were identified through Venn diagram analysis (Fig 2C). S1 Table provides details of the identified DEGs.

### WGCNA

**Fig 3:**
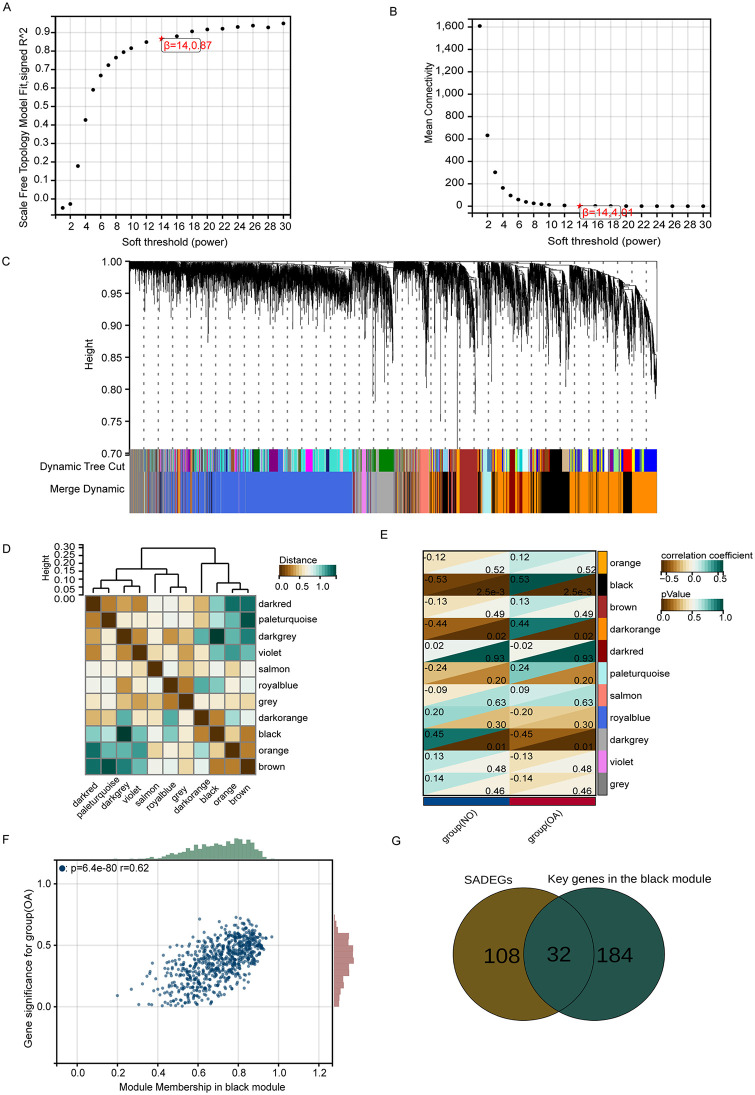
WGCNA. **(A, B)** Determination of the optimal soft threshold. **(C)** Eleven gene modules were obtained by hierarchical clustering. **(D)** Cluster plot of module feature vectors. **(E)** Heatmap of associations between gene modules and OA phenotypes. **(F)** Scatterplot showing the correlation between gene significance (GS) and module membership (MM) in the black module. **(G)** Venn diagram showing the intersection of SADEGs and key genes in the black module.

We performed WGCNA analysis on the training set. A soft threshold of 14 was selected as the optimal soft threshold (R^2^ = 0.87) to establish the gene co-expression network (Fig 3A and 3B). An unsigned network was used, and modules were identified with a minimum module size of 30, deepSplit = 3, and a merge cut height of 0.25. Eleven gene modules were obtained by hierarchical clustering (Fig 3C). In the gene-OA trait association analysis, the black module showed the strongest association with OA traits, with a correlation coefficient of 0.53 and *p* = 2.5e-3 (Fig 3D and 3E). In addition, GS was strongly correlated with MM within the black module (cor = 0.62; *p* = 6.4e-8) (Fig 3F). A total of 216 genes strongly associated with OA were selected from the black module, and 32 OA-SADEGs were obtained by intersecting these genes with SADEGs (Fig 3G).

### Functional enrichment analysis of OA-SADEGs

**Fig 4:**
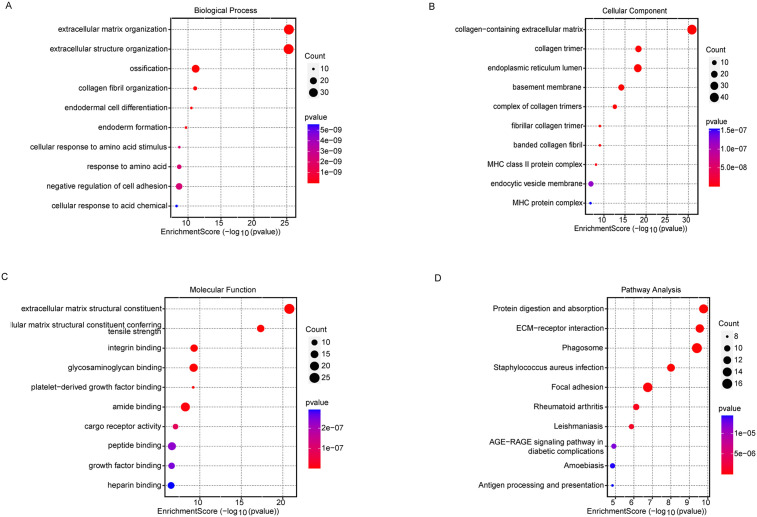
Functional enrichment analysis of OA-SADEGs. **(A)** Bubble plot of GO terms in the BP category. **(B)** Bubble plot of GO terms in the CC category. **(C)** Bubble plot of GO terms in the MF category. **(D)** Bubble plot of KEGG pathways.

To explore the potential biological functions and pathways associated with OA-SADEGs, we used the R package “clusterProfiler” to perform GO and KEGG enrichment analysis. Enrichment significance was evaluated using the Benjamini–Hochberg method, and adjusted *p* < 0.05 was considered statistically significant. In the biological process (BP) category, OA-SADEGs were mainly enriched in extracellular matrix organization, extracellular structure organization, ossification, and collagen fibril organization (Fig 4A). In the cellular component (CC) category, OA-SADEGs were mainly enriched in the collagen-containing extracellular matrix, collagen trimer, endoplasmic reticulum lumen, basement membrane, and collagen trimer complex (Fig 4B). In the molecular function (MF) category, OA-SADEGs were mainly enriched in extracellular matrix structural constituent, extracellular matrix structural constituent conferring tensile strength, integrin binding, and glycosaminoglycan binding (Fig 4C). The pathways in which they were mainly involved were protein digestion and absorption, ECM-receptor interaction, phagosome, staphylococcus aureus infection, focal adhesion and antigen processing and presentation (Fig 4D). Detailed results for GO and KEGG enrichment of OA-SADEGs are shown in S2-S5 Tables.

### Identification of hub genes

**Fig 5:**
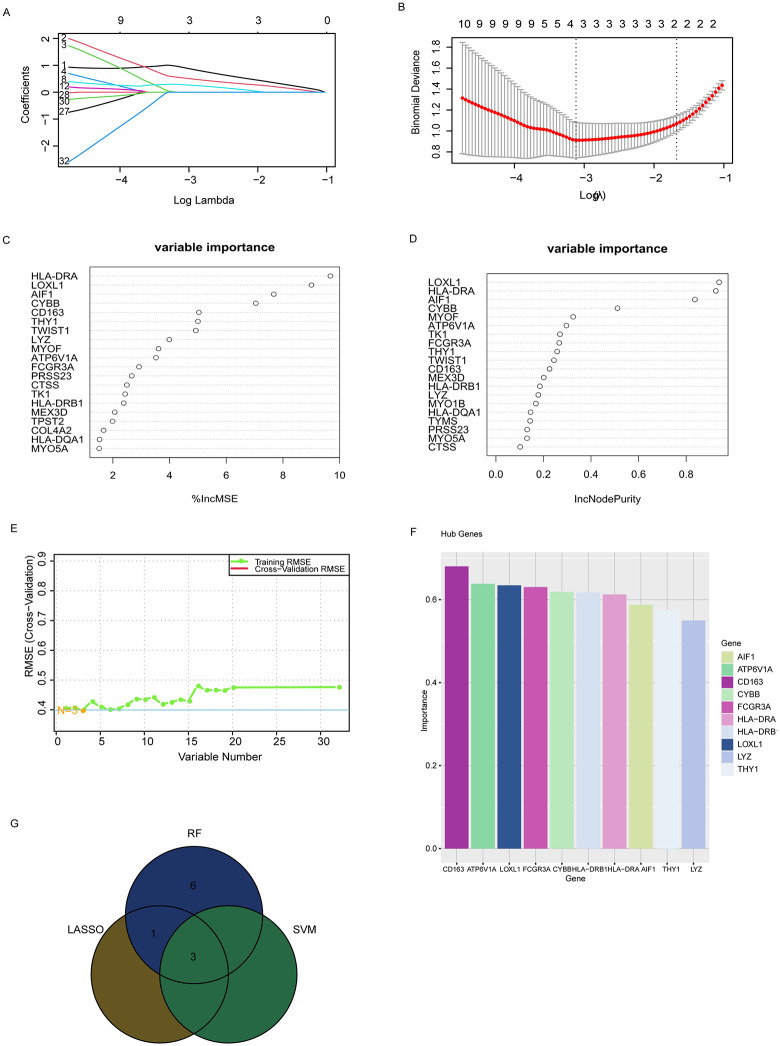
Identification of hub genes. **(A)** Ten-fold cross-validation of the LASSO model for selecting the optimal λ value. **(B)** LASSO coefficient analysis. **(C, D)** Ranking of gene importance by two RF metrics. **(E, F)** Ten-fold cross-validation with five replicates to identify the optimal feature subset with the lowest RMSE. **(G)** Venn diagram showing the intersection of genes selected by LASSO, RF, and SVM-RFE to identify the final hub genes.

In the LASSO analysis, four genes, LOXL1, HLA-DRA, CYBB, and ATP6V1A, were selected at the optimal λ value indicated by the vertical dashed line (Fig 5A and 5B). Gene importance was evaluated by RF using two different metrics. The top ten genes ranked by IncNodePurity were selected for further comparison (Fig 5C and 5D). LOXL1, HLA-DRA, and CYBB were selected by SVM-RFE when the RMSE reached its minimum value (Fig 5E and 5F). The three hub genes—LOXL1, HLA-DRA, and CYBB—were obtained by intersecting the genes identified by LASSO, RF, and SVM-RFE (Fig 5G). Detailed results from the three machine learning algorithms are shown in S6 Table.

### Expression and discriminative ability of hub genes

**Fig 6:**
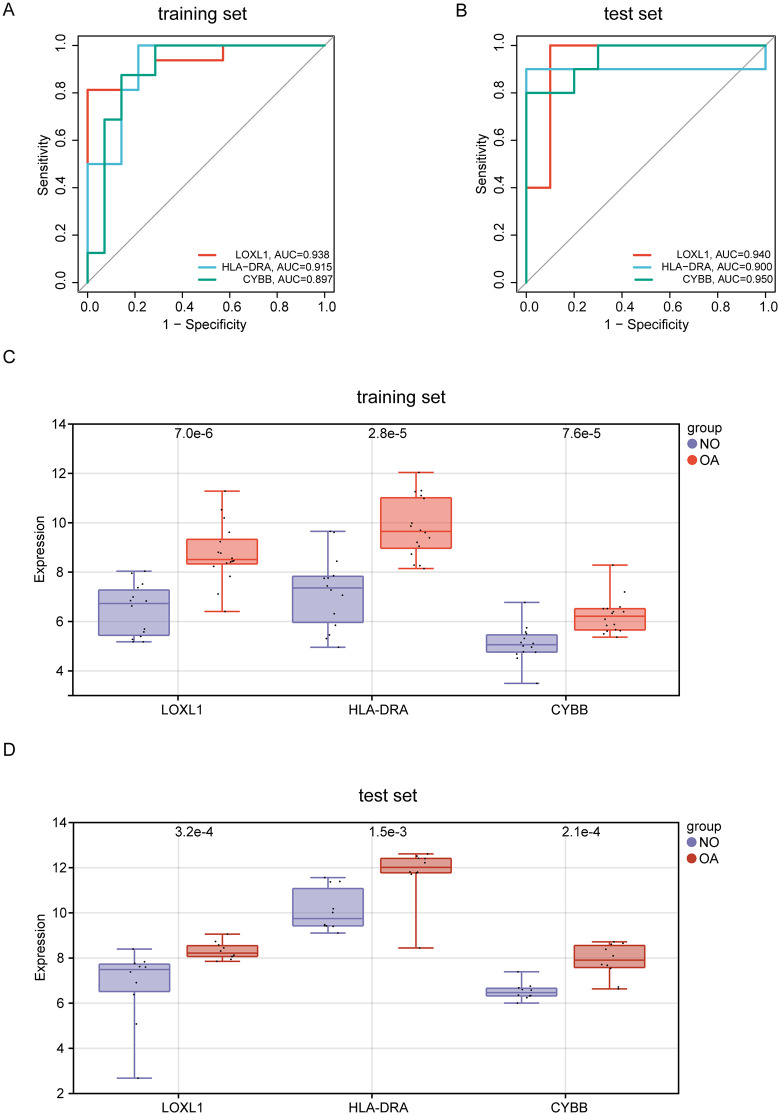
Evaluation of the expression and discriminative ability of hub genes. **(A)** ROC curve analysis of hub genes in the training set. **(B)** ROC curve analysis of hub genes in the test set. **(C)** Box plot of hub gene expression in Normal and OA samples in the training set. **(D)** Box plot of hub gene expression in Normal and OA samples in the test set. *p < 0.05; **p < 0.01; ***p < 0.001.

The AUCs of the three hub genes were all above 0.7 in both the training and test sets (Fig 6A and 6B), suggesting that LOXL1, HLA-DRA, and CYBB had potential discriminative ability between OA and control samples. In addition, LOXL1, HLA-DRA, and CYBB showed significant expression differences between OA and control samples in both the training and test sets. All three genes were consistently upregulated in OA samples compared with control samples in both the training and test sets (Fig 6C and 6D). Consequently, LOXL1, HLA-DRA, and CYBB were identified as candidate hub genes. Hub gene expression values in the training and test sets are shown in S7 and S8 Tables.

### xCell immune infiltration analysis

**Fig 7:**
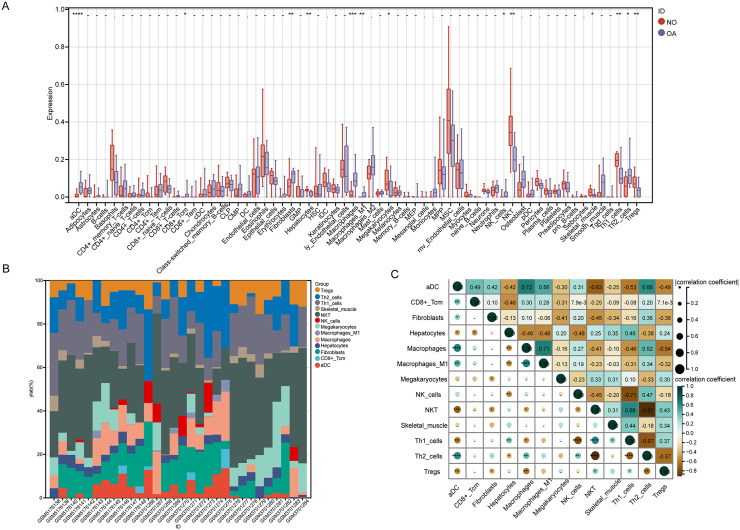
xCell immune infiltration analysis of OA-SADEGs. (A) Box plot of the 64 immune cell types. (B) Relative proportions of 13 differentially enriched immune cell types. (C) Correlation heatmap showing correlations among the 13 differentially enriched immune cell types.

**Fig 8:**
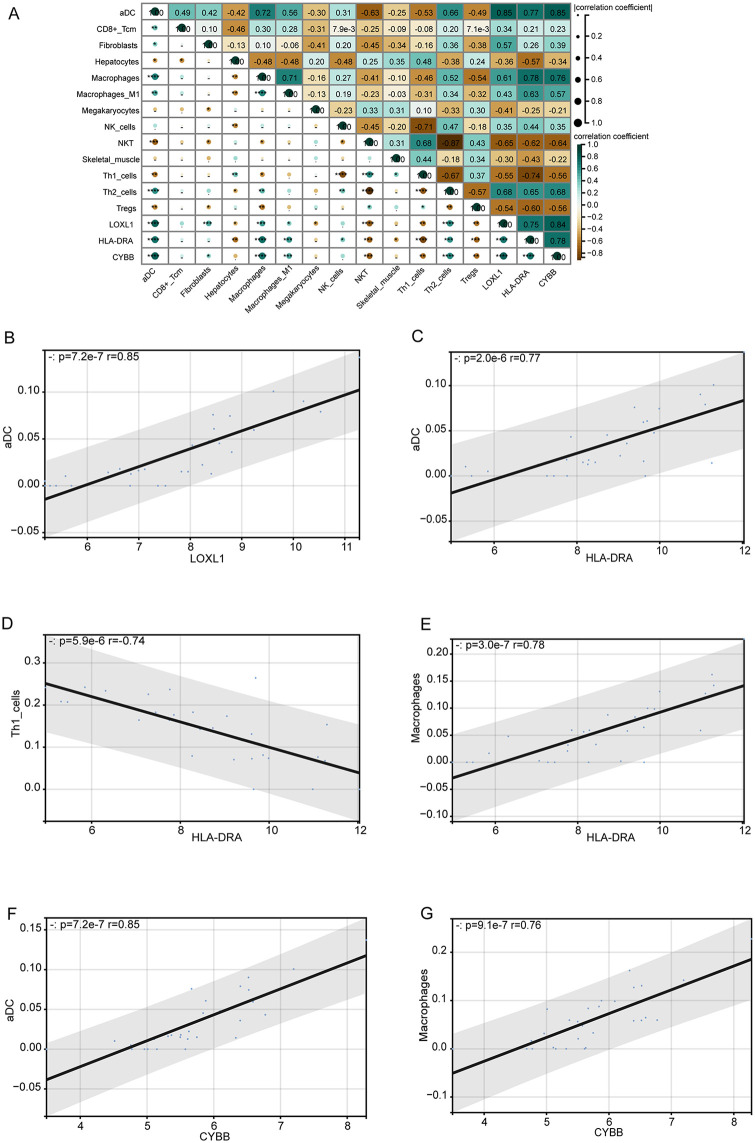
Correlation analysis between hub genes and immune cell infiltration scores. **(A)** Heatmap of the association between hub genes and 13 immune cell infiltration scores. **(B)** LOXL1 was positively correlated with aDC (*p* = 7.2e-7, r = 0.85). **(C)** HLA-DRA was positively correlated with aDC (*p* = 2.0e-6, r = 0.77). **(D)** HLA-DRA was negatively correlated with Th1 cells (*p* = 5.9e-6, r = −0.74). **(E)** HLA-DRA was positively correlated with macrophages (*p* = 3.0e-7, r = 0.78). **(F)** CYBB was positively correlated with aDC (*p* = 7.2e-7, r = 0.85). **(G)** CYBB was positively correlated with macrophages (*p* = 9.1e-7, r = 0.76).

Pathway and hub gene analyses suggested that sex hormone-associated genes were related to immune-related pathways and features. To explore the immune-related characteristics of sex hormone-associated genes in OA, we conducted xCell immune infiltration analysis of OA-SADEGs. The relative infiltration scores of 64 immune and stromal cell types were visualized across groups, and 13 differentially enriched cell types were identified (Fig 7A): aDC, CD8 + Tcm, fibroblasts, hepatocytes, macrophages, macrophages M1, megakaryocytes, NK cells, NKT, skeletal muscle, Th1 cells, Th2 cells, and Tregs. We calculated the relative proportions of the 13 cell types to illustrate their distribution across different samples (Fig 7B). The correlation plot was used to illustrate correlations among the estimated cell infiltration scores. aDC was positively correlated with macrophages, while macrophages were positively correlated with macrophages M1. NK cells were negatively correlated with Th1 cells, and NKT cells were negatively correlated with Th2 cells (Fig 7C). Furthermore, LOXL1 was strongly correlated with aDC; HLA-DRA with aDC, Th1 cells, and macrophages; and CYBB with aDC and macrophages (Fig 8). Re-analysis of an independent external dataset using xCell showed generally consistent trends in the major immune infiltration patterns identified in the training set (S2 Fig and S3 Fig).

### qRT-PCR validation

**Fig 9:**
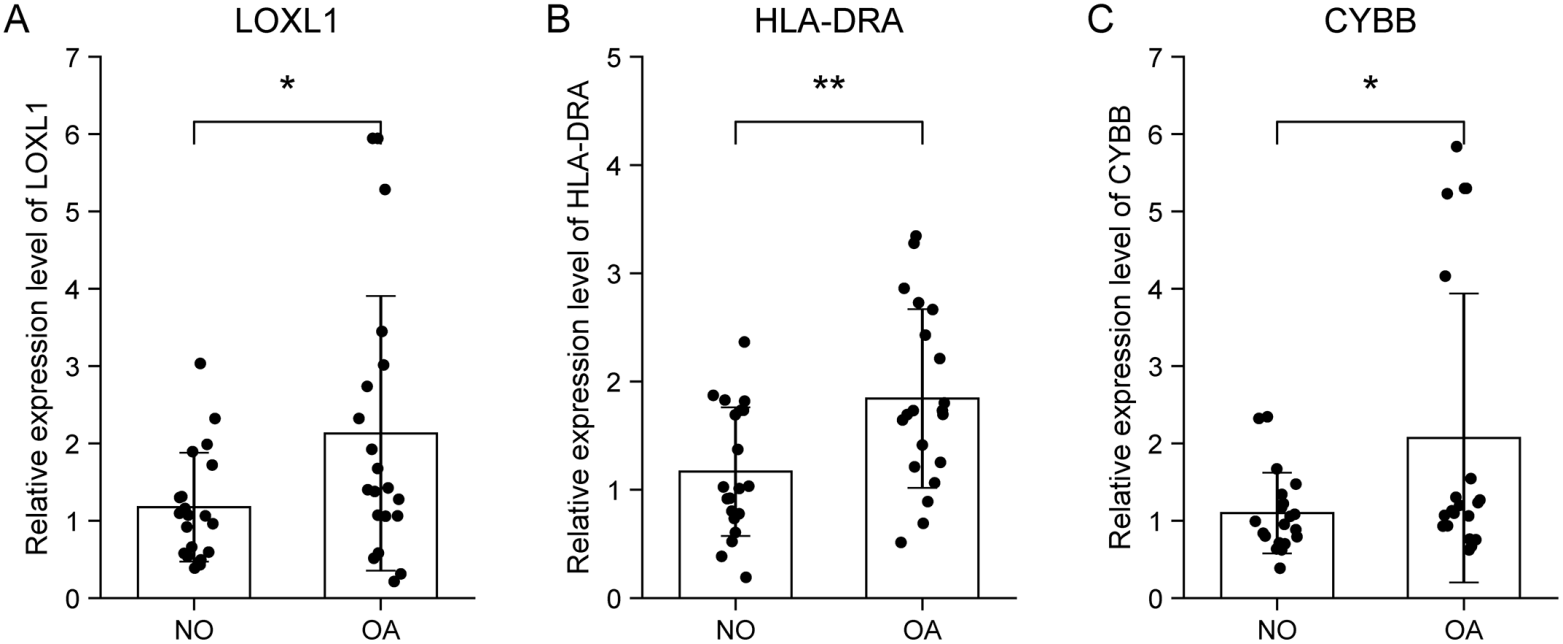
qRT-PCR. (A-C) LOXL1, HLA-DRA, and CYBB were significantly upregulated in peripheral blood samples from OA patients compared with the NO group. *p < 0.05, **p < 0.01.

The qRT-PCR validation samples included 20 OA patients and 20 controls. The proportion of females was 80.0% in the OA group and 85.0% in the control group (*p* = 1.000). The mean age was 63.05 ± 10.04 years in the OA group and 63.30 ± 10.71 years in the control group (*p* = 0.940), and the mean BMI was 24.23 ± 3.28 kg/m² and 24.70 ± 4.12 kg/m², respectively (*p* = 0.692). These results indicated that the baseline characteristics were comparable between the two groups. The relative expression levels of LOXL1, HLA-DRA, and CYBB in peripheral blood samples from OA patients and controls were measured by qRT-PCR. The results indicated that LOXL1, HLA-DRA, and CYBB were significantly upregulated in OA compared to the control group (Fig 9A-C). Primer sequences for each gene are listed in S9 Table.

## Discussion

Our findings identified three candidate hub genes (LOXL1, HLA-DRA, and CYBB), along with several enriched pathways and immune-related cell features that may be associated with OA. GO enrichment analysis showed that these genes were mainly enriched in biological processes such as extracellular matrix (ECM) organization and ossification. This enrichment is notable because ECM homeostasis is central to cartilage integrity and joint remodeling in OA [13]. Ossification contributes to the formation of osteophytes, which in turn leads to joint stiffness and pain in patients with OA [14]. Among the cellular components, the identified genes were mainly enriched in the collagen-containing extracellular matrix and collagen trimer, suggesting a potential association with cartilage structural components. Regarding molecular functions, these genes were mainly enriched in extracellular matrix structural constituents and integrin binding. Integrins mediate interactions between cells and the ECM and regulate cell signaling, adhesion, and migration, which may contribute to OA progression [15]. KEGG analysis showed enrichment in pathways related to protein digestion and absorption, ECM–receptor interaction, phagosome, and antigen processing and presentation, suggesting potential links with matrix remodeling and immune-related processes in OA. Enrichment of the protein digestion and absorption pathway may reflect protease-related extracellular matrix degradation in OA. Collagen, a major extracellular matrix component, undergoes degradation, which is a key feature of OA [16]. Matrix metalloproteinase-13 (MMP-13) is a key collagenase whose overexpression contributes to cartilage erosion and degeneration and may thereby exacerbate OA [17]. Phagocytosis is crucial for removing apoptotic cells and pathogen-related molecules from the joints [18]. In OA, macrophages and other immune cells phagocytose apoptotic chondrocytes, influencing disease progression [19]. Enrichment of the antigen processing and presentation pathway suggests a potential association between immune-related processes and OA progression. An imbalance in immune responses may contribute to chronic inflammation and cartilage destruction [20]. Reduced estrogen levels may promote macrophage secretion of osteoclast-related cytokines and osteoclast activation, thereby potentially contributing to OA progression [21].

LOXL1 (lysyl oxidase-like 1), a member of the lysyl oxidase family, plays a critical role in connective tissue biogenesis. The prototypic member of this family is essential for ECM formation. LOXL1 has been reported to promote the cross-linking of collagen and elastin through its lysyl oxidase activity, thereby enhancing ECM strength and stability. In OA, LOXL1 has been reported to be upregulated and associated with cartilage degeneration and increased inflammation. Specifically, LOXL1 may participate in synovial inflammation through the PI3K/AKT signaling pathway [22]. Treatment with inhibitors such as ZSTK474 has been reported to reduce inflammatory responses associated with LOXL1 and PI3K/AKT signaling [23]. Although direct evidence that sex hormones regulate LOXL1 in OA is lacking, sex hormones may be indirectly linked to LOXL1-associated ECM remodeling in OA. In fibrotic contexts, E2 has been shown to inhibit the TGF-β1/Smad3 pathway and reduce LOXL1-mediated ECM hypercross-linking, thereby delaying fibrosis [24,25]. HLA-DRA (major histocompatibility complex, class II, DR alpha) encodes the alpha chain of the HLA-DR class II molecule. Proteins encoded by HLA-DRA play a crucial role in antigen presentation, primarily by presenting antigens to CD4 + T cells and thereby initiating immune responses. Elevated HLA-DRA expression in OA has been reported to be associated with increased immune cell infiltration, including macrophages and T cells, and higher levels of proinflammatory cytokines, such as IL-1β and TNF-α [26]. Sex hormones, such as estrogen, have been shown to modulate the expression of HLA class II molecules in immune cells. Changes in sex hormone levels may indirectly influence HLA-DRA-mediated inflammation in OA via the antigen presentation pathway, though this hypothesis requires experimental validation [27]. CYBB (cytochrome b-245 β chain), also referred to as NADPH oxidase 2 (NOX2), functions as a key subunit of the NADPH oxidase complex, generating reactive oxygen species (ROS), and its overexpression contributes to oxidative stress. Oxidative stress is involved in OA pathogenesis by inducing chondrocyte apoptosis, promoting matrix degradation, enhancing inflammation, impairing cellular function, and contributing to matrix sclerosis [28,29]. NOX2-derived oxidative stress may exacerbate MMP-mediated cartilage degradation, potentially contributing to tissue destruction in OA [30]. Oxidative stress is associated with homocysteine, a well-established biomarker linked to ROS production and redox imbalance. Previous studies have shown that sex hormones may modulate homocysteine levels, which may in turn influence NOX-mediated ROS production [31]. Additionally, evidence suggests that the antioxidant properties of E2 help protect cartilage against ROS-induced damage [32].

In this study, we identified 13 differentially enriched immune cell types and observed significant associations between hub genes and selected immune cells. These findings provide insights into potential associations among sex hormone-associated genes, immune-related features, and OA. Macrophages, particularly the M1 subtype, have been implicated in synovial inflammation and cartilage degradation by producing pro-inflammatory cytokines, such as TNF-α and IL-1β, and ROS via NOX2 activity [33]. Several studies have shown that the M1/M2 macrophage ratio is significantly higher in patients with knee OA than in controls, and this ratio is positively correlated with Kellgren-Lawrence grade [34]. The positive correlation between macrophages and aDC may suggest a coordinated pro-inflammatory immune pattern in OA. An imbalance between Th1 and Th2 cells has been reported in OA, with Th1 cells secreting IFN-γ and TNF-α and potentially promoting inflammation and joint degeneration, whereas Th2 cells are generally associated with anti-inflammatory responses [35]. Natural killer (NK) cells are innate immune cells that have been implicated in OA-related inflammation, partly through the production of IL-6 and TNF-α and recruitment via the CXCL10/CXCR3 axis [36,37]. They may also eliminate activated T cells through LFA-1-, NKG2D-, and TRAIL-mediated mechanisms and modulate adaptive immunity by regulating T cell activation [36]. The interaction between NK and T cells may be involved in immune infiltration and OA progression. Sex hormones may also be involved in OA-related immune regulation by influencing key immune cells. Estrogen has been reported to enhance anti-inflammatory macrophage responses, including cytokine production, chemotaxis, and phagocytosis [38]. It has also been reported to modulate NK cell activity by reducing NK cell infiltration, cytotoxicity, and proliferation, which may contribute to reduced inflammation [39]. Additionally, estrogen may modulate the Th1/Th2 balance by promoting Th2 cell cytokine secretion, thereby contributing to immune regulation [40]. Androgen has been shown to promote macrophage migration and the production of anti-inflammatory cytokines [41]. Correlation analysis showed that all three hub genes were strongly associated with immune cell infiltration, suggesting potential links between sex hormone-associated genes and immune alterations in OA. Furthermore, the potential role of sex hormones in modulating immune responses may provide a theoretical context for understanding OA-related immune alterations, but the underlying mechanisms require further experimental validation.

Although this study identified candidate sex hormone-associated genes in OA through bioinformatics analysis and qRT-PCR validation, several limitations remain. First, the links between LOXL1, HLA-DRA, and CYBB and sex hormone regulation in OA remain indirect and associative. The initial sex hormone-associated gene list was derived from the broadly annotated GeneCards database without gene-by-gene manual curation and may include genes with indirect or weak sex hormone-related associations. Therefore, the findings should be interpreted as candidate associations rather than direct evidence of sex hormone-mediated regulation. Second, clinical validation still has limitations. Direct estrogen and testosterone measurements were not performed, and detailed endocrine-related characteristics were not fully available. Therefore, the relationship between hormone status and candidate gene expression could not be directly assessed. Future studies should include larger validation samples with direct hormone measurements and more detailed endocrine information. Third, qRT-PCR validation was performed using peripheral blood samples rather than joint tissues. Therefore, the results should be interpreted as supportive biomarker evidence, rather than biological confirmation of tissue-level mechanisms or direct validation of cartilage or synovial transcriptomic findings. Future studies using joint tissues, synovial fluid, or paired blood–tissue samples are needed. Fourth, xCell immune infiltration analysis was based on computational estimation rather than direct biological validation, and experimental validation of the inferred immune cell patterns was not performed. In addition, functional experiments, such as gene knockdown or overexpression, chondrocyte or synoviocyte assays, ECM degradation analysis, inflammatory signaling assays, oxidative stress assays, or in vivo validation, were not performed. Therefore, LOXL1, HLA-DRA, and CYBB should be regarded as candidate associated genes or candidate markers rather than functionally validated regulators.

## Conclusion

In summary, by integrating bioinformatics analysis with qRT-PCR validation, this study systematically explored candidate sex hormone-associated genes in OA and identified three candidate hub genes (LOXL1, HLA-DRA, and CYBB), which exhibited significant upregulation in both external datasets and peripheral blood samples. Furthermore, immune infiltration analysis suggested immune-related features associated with OA, providing associative evidence for the potential links among sex hormone-associated genes, immune alterations, and OA.

## Supporting information

S1 Fig Evaluation of batch effect removal before and after batch correction. (A) UMAP plot before batch correction. (B) UMAP plot after batch correction. The separation between datasets was markedly reduced after batch correction, indicating that batch effects were effectively alleviated.
(TIF)

S2 Fig External assessment of immune infiltration patterns and hub gene–immune cell associations using xCell. (A) Differential immune cell infiltration patterns between the NO and OA groups in the independent external dataset, as estimated by xCell. (B) Correlation heatmap showing the associations between hub genes and major immune cell populations in the independent external dataset.
(TIF)

S3 Fig External assessment of representative hub gene–immune cell correlations. (A–F) Representative correlations between hub genes and major immune cell populations in the independent external dataset, including CYBB–macrophages, CYBB–aDC, HLA-DRA–macrophages, HLA-DRA–Th1 cells, HLA-DRA–aDC, and LOXL1–aDC.
(TIF)

S1 Table List of differentially expressed genes. (XLSX)

S2 Table GO biological process enrichment analysis of OA-SADEGs. (XLSX)

S3 Table GO cellular component enrichment analysis of OA-SADEGs. (XLSX)

S4 Table GO molecular function enrichment analysis of OA-SADEGs. (XLSX)

S5 Table KEGG pathway enrichment analysis of OA-SADEGs. (XLSX)

S6 Table Hub gene identification results from LASSO, SVM-RFE, and random forest analyses. (XLSX)

S7 Table Expression values of hub genes in the training set. (XLSX)

S8 Table Expression values of hub genes in the test set. (XLSX)

S9 Table Primer sequences used for qRT-PCR validation. (XLSX)
