# Supplementary figures and images for: Identification of candidate sex hormone-associated genes and immune infiltration characteristics in osteoarthritis based on bioinformatics analysis and machine learning
Source: PLoS One. 2026 Jun 12;21(6):e0351556. doi: 10.1371/journal.pone.0351556 (PMC13262821; doi:10.1371/journal.pone.0351556)

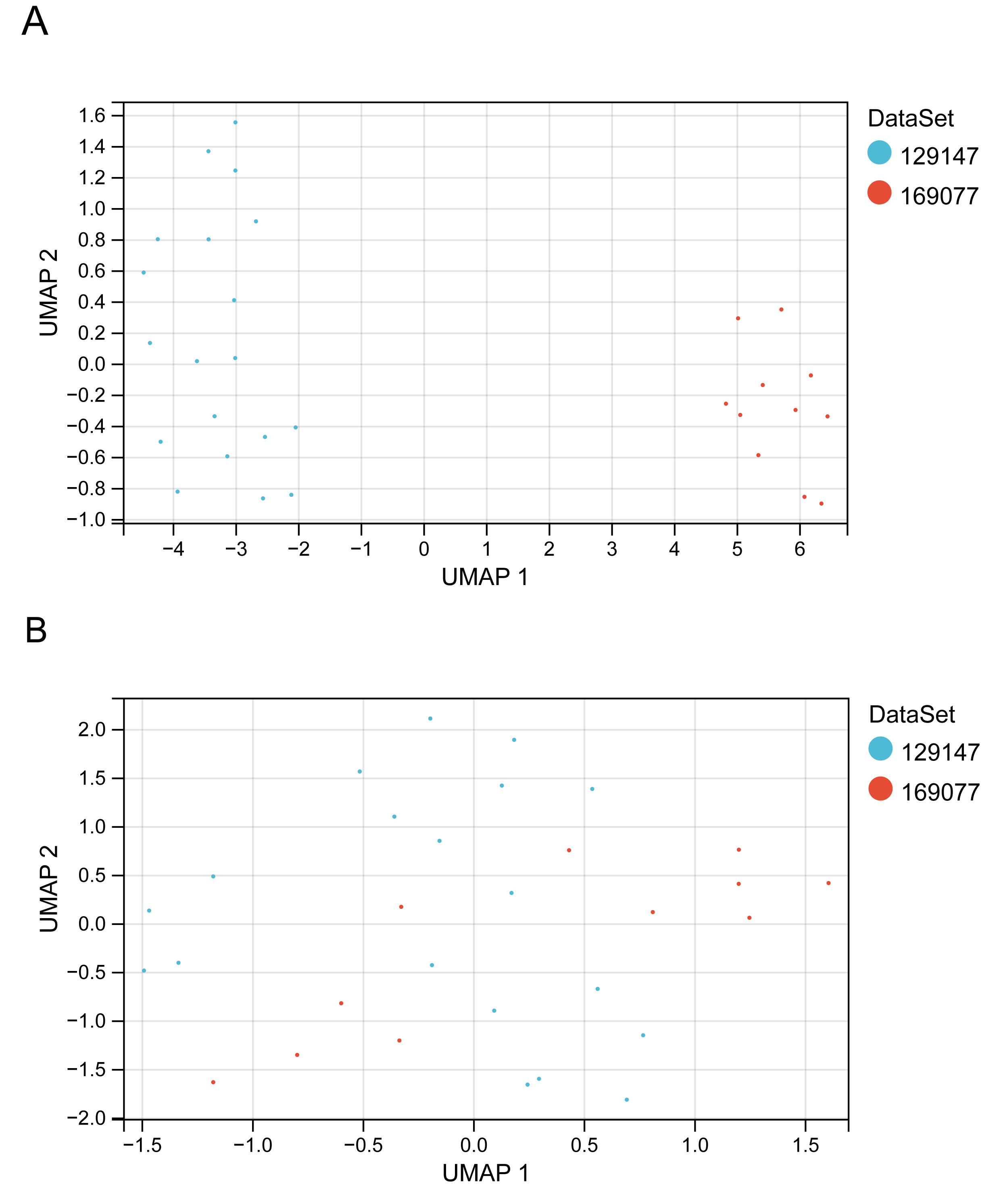

Supplement: S1 Fig — (A) UMAP plot before batch correction. (B) UMAP plot after batch correction. The separation between datasets was markedly reduced after batch correction, indicating that batch effects were effectively alleviated. (TIF) [file pone.0351556.s001.tif]

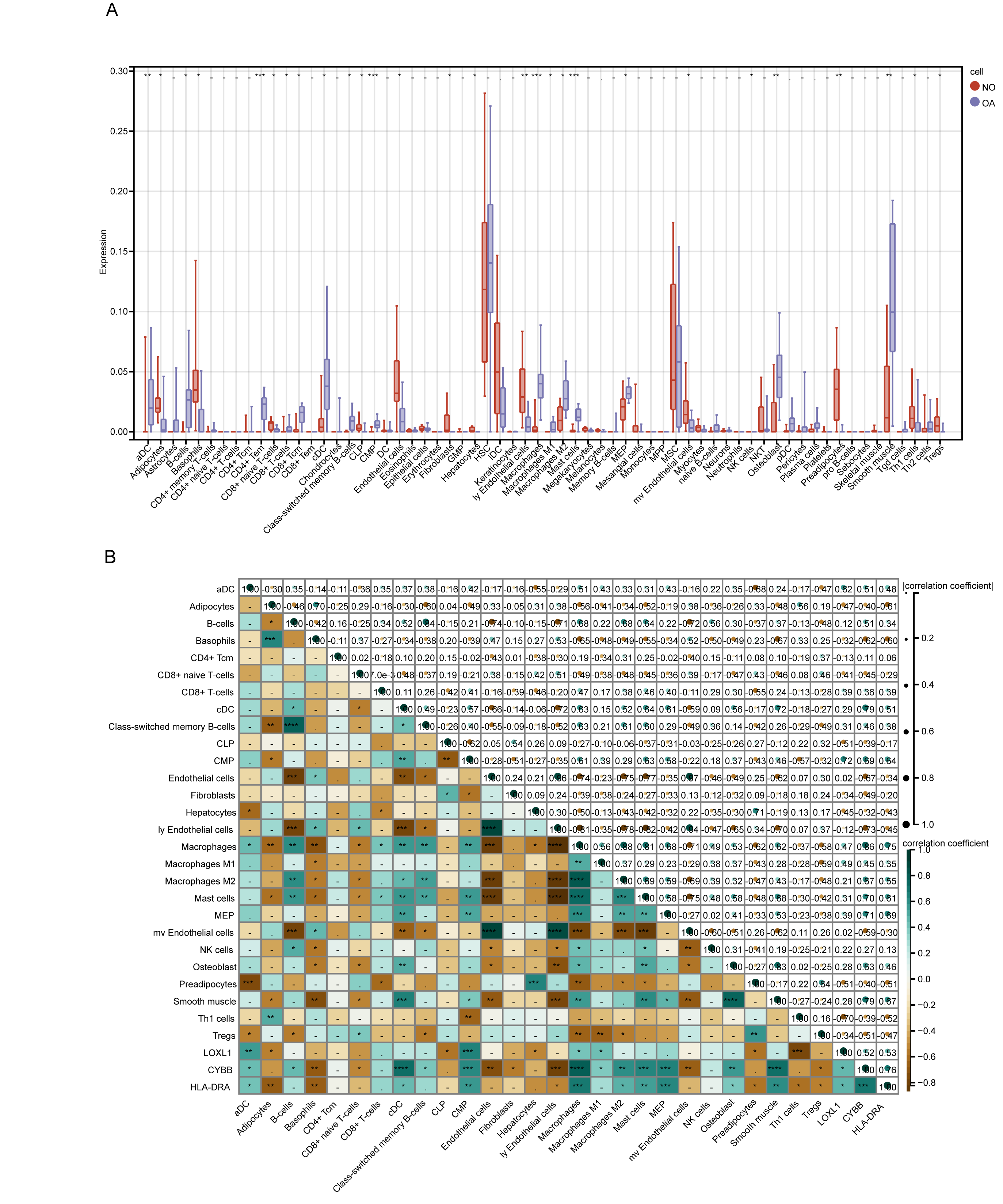

Supplement: S2 Fig — (A) Differential immune cell infiltration patterns between the NO and OA groups in the independent external dataset, as estimated by xCell. (B) Correlation heatmap showing the associations between hub genes and major immune cell populations in the independent external dataset. (TIF) [file pone.0351556.s002.tif]

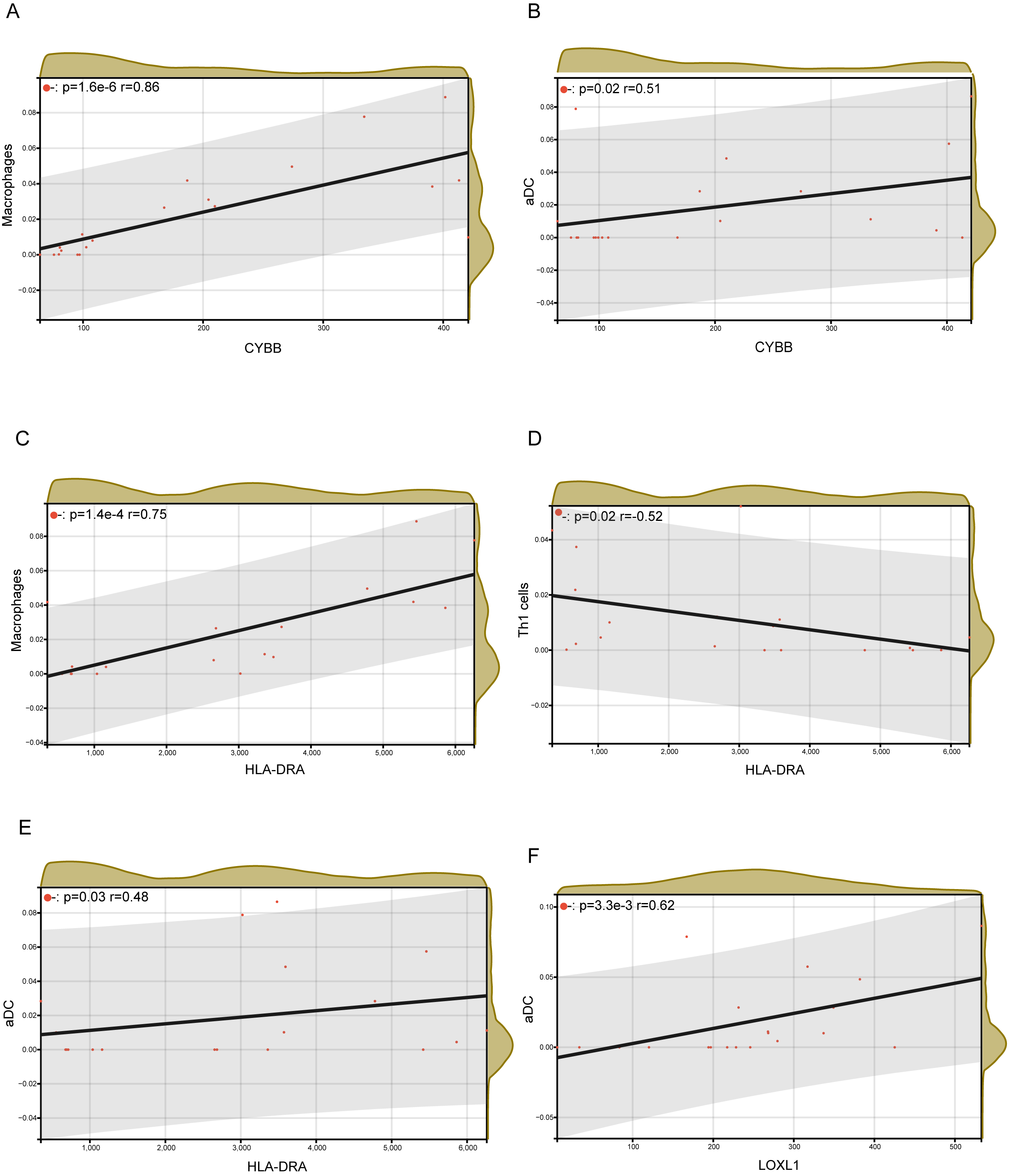

Supplement: S3 Fig — (A–F) Representative correlations between hub genes and major immune cell populations in the independent external dataset, including CYBB–macrophages, CYBB–aDC, HLA-DRA–macrophages, HLA-DRA–Th1 cells, HLA-DRA–aDC, and LOXL1–aDC. (TIF) [file pone.0351556.s003.tif]
